# Dietary Glycotoxins Impair Hepatic Lipidemic Profile in Diet-Induced Obese Rats Causing Hepatic Oxidative Stress and Insulin Resistance

**DOI:** 10.1155/2019/6362910

**Published:** 2019-06-25

**Authors:** C. Neves, T. Rodrigues, J. Sereno, C. Simões, J. Castelhano, J. Gonçalves, G. Bento, S. Gonçalves, R. Seiça, M. R. Domingues, M. Castelo-Branco, P. Matafome

**Affiliations:** ^1^Institute of Physiology and iCBR, Faculty of Medicine, University of Coimbra, Portugal; ^2^Institute for Nuclear Sciences Applied to Health (CIBIT, ICNAS), University of Coimbra, Coimbra, Portugal; ^3^Departamento de Química & CESAM & ECOMARE, Universidade de Aveiro, Aveiro, Portugal; ^4^Instituto Politécnico de Coimbra, Coimbra Health School, Department of Complementary Sciences, ESTeSC, Coimbra, Portugal

## Abstract

Nonalcoholic fatty liver disease (NAFLD) is caused by excessive liver lipid accumulation, but insulin resistance is specifically associated with impaired lipid saturation, oxidation, and storage (esterification), besides increased de novo lipogenesis. We hypothesized that dietary glycotoxins could impair hepatic lipid metabolism in obesity contributing to lipotoxicity-driven insulin resistance and thus to the onset of nonalcoholic steatohepatitis (NASH). In diet-induced obese rats with methylglyoxal-induced glycation, magnetic resonance spectroscopy, mass spectrometry, and gas chromatography were used to assess liver composition in fatty acyl chains and phospholipids. High-fat diet-induced obesity increased liver lipid fraction and suppressed de novo lipogenesis but did not change fatty acid esterification and saturation or insulin sensitivity. Despite a similar increase in total lipid fraction when supplementing the high-fat diet with dietary glycotoxins, impairment in the suppression of de novo lipogenesis and decreased fatty acid unsaturation and esterification were observed. Moreover, glycotoxins also decreased polyunsaturated cardiolipins and caused oxidative stress, portal inflammation, and insulin resistance in high-fat diet-induced obese rats. Dietary glycated products do not change total lipid levels in the liver of obese rats but dramatically modify the lipidemic profile, leading to oxidative stress, hepatic lipotoxicity, and insulin resistance in obesity and thus contribute to the onset of NASH.

## 1. Introduction

Obesity, body mass index (BMI) > 30 kg/m^2^, is a major cause of morbidity and mortality, associated with an increased risk of metabolic syndrome and type 2 diabetes mellitus (T2DM) [1]. Diabetes and obesity are closely correlated with hepatic steatosis and consequently nonalcoholic fatty liver disease (NAFLD), a pathology characterized by excessive fat accumulation in liver, namely, free fatty acids (FFA), also called nonesterified fatty acids (NEFA) [2, 3]. Liver fat accumulation depends on FFA flux from lipolysis, dietary absorption, and *de novo* lipogenesis as well as decreased fatty acid oxidation [3]. Lipotoxicity, a process known as the activation of inflammatory mechanisms by NEFA, induces insulin resistance, which increment the risk for glucose dysmetabolism and T2DM [4, 5]. In particular, saturated fatty acids and linoleic acid (18 : 2) were reported to induce inflammation and mitochondrial dysfunction, increasing reactive oxygen species (ROS), lipid peroxidation, and further lipid deposition [4]. Liver inflammation may be an initial protective mechanism against excessive lipid and glucose uptake, but when chronically activated, it conducts to the onset of nonalcoholic steatohepatitis (NASH), decreasing insulin and AMPK signaling and glucose and lipid uptake [4, 6]. Although initially considered the liver manifestation of the metabolic syndrome, NAFLD is now believed to cause it, due to the development of insulin resistance, which contributes to systemic glucose and lipid dysmetabolism [2]. However, the mechanisms involved in the development of insulin resistance are not fully known.

Obese and diabetic patients have increased blood levels of advanced glycation-end products (AGE) and the reactive dicarbonyl methylglyoxal (MG) (reviewed by [7]). MG is a glycating agent which reacts nonenzymatically with proteins, lipids, and DNA leading to AGE formation. Interestingly, the normalization of blood glucose in diabetic patients does not completely restore MG levels, suggesting that persistent MG formation occurs due to acquired errors in metabolism, leading to the concept of disrupted “metabolic memory” [7]. In addition, sugar-rich and processed foods have high MG levels, mainly due to its formation from glucose and fructose. Despite the fact that its pathophysiology significance is not yet fully understood, several studies have suggested reduction of dietary AGE as a strategy to prevent diabetes-like complications [8, 9]. MG has been implicated in various pathological conditions such as insulin resistance, *β*-cell failure, and diabetic macro- and microvascular complications. MG is detoxified into D-lactate by the glyoxalase system (GLO-1 and GLO-2). GLO-1 overexpression was shown to prevent glucose-induced ROS production and the development of diabetic complications [10].

Thus, our aim was to assess the modifications of liver lipid metabolism caused by dietary glycated products in obesity-associated fatty livers, as well as their role in the development of insulin resistance and glucose intolerance. Thus, we developed a model of hepatic steatosis by feeding normal rats with a triglyceride-enriched diet and supplemented a group of them with MG-derived glycation products. Magnetic resonance spectroscopy (MRS), high-performance liquid chromatography (HPLC), and mass spectrometry (MS) were used to analyse liver lipidemic profile.

## 2. Research Design and Methods

### 2.1. Reagents and Antibodies

Salts and organic solvents used in solution preparations were purchased from Fisher Scientific (Leicestershire, UK), Sigma Chemicals (USA), or Merck Darmstadt (Germany), with the highest grade of purity commercially available. Antibodies used were targeted to AMPK and (Thr172)AMPK (#2532, #2535, Cell Signaling, USA); F4/80, GLUT2, ACC, (Ser79)ACC, and (Tyr1163)IR*β* (ab74383, ab54460, ab72046, ab68191, and Ab60946, Abcam, UK); IR*β* (sc-57342, Santa Cruz Biotechnology, USA) argpyrimidine (AGE06B, Nordic MUbio, Netherlands); and MG-H1 (HM5017, Hycult Biotech, Netherlands). Calnexin was used as loading control (AB0037, SICGEN, Portugal).

### 2.2. Animal Maintenance

Wistar rats from our breeding colonies (Faculty of Medicine, University of Coimbra) were kept under standard conditions [11, 12]. The experimental protocol was approved by the local Institutional Animal Care and Use Committee (ORBEA-FMUC 3/15) and Portuguese Veterinary Authority (DGAV). All the procedures were performed by licensed users of the Federation of Laboratory Animal Science Associations (FELASA).

### 2.3. Experimental Groups

Male Wistar rats were randomly divided into four groups (*n* = 8/group): (1) control (Ct) with standard diet A03 (5% triglycerides and 45% carbohydrates, SAFE, France), (2) methylglyoxal group (MG) with standard diet and MG administration, (3) high-fat diet-fed group (HFD), and (4) high-fat diet group with MG supplementation (HFDMG).

### 2.4. Diet and MG Administration

High-fat (HF) diet (40% triglycerides and 10% carbohydrates, 231 HF, SAFE, France) was administered during 18 weeks (8 to 12 months old). In order to increase dietary glycated products, daily MG (75 mg kg^−1^) was administered orally as described before [12–15]. This protocol increases the reaction of MG with food components and consequent formation of MG adducts and AGE, which are then absorbed as exogenous dietary AGE. We have previously shown that it results in plasma and tissue MG and AGE levels similar to diabetic rats [12–14], being a more physiologic protocol than i.p. or subcutaneous injection, which can result in supraphysiological doses.

### 2.5. Body Weight and Glycemic Profile

In overnight (18 h) fasted rats, body weight was recorded and glycemia (fasting and 1 and 2 hours after i.p. glucose administration; 1.8 g kg^−1^; IPGTT) was measured in the tail vein.

### 2.6. Magnetic Resonance Spectroscopy

High-resolution 1H magnetic resonance spectroscopy (1H-MRS) of liver tissues (noninvasive technique) coupled with principal component analysis was performed using a BioSpec 9.4 T MRI scanner (Bruker BioSpin, Ettlingen, Germany). Rats (*n* = 6/group) were kept anesthetized by isoflurane (2-3%) with 100% O_2_ with body temperature (37°C) and respiration monitoring (SA Instruments SA, Stony Brook, USA). Water-suppressed 1H NMR liver lipid spectrums were analysed (apodization, fast Fourier transform, and peak fitting) by an automatic peak-fitting procedure (LCModel) to determine the area of each peak. Homemade software implemented in Matlab (v2013a, MathWorks) was used to obtain hepatic lipid signals corrected for signal decay due to spin-spin relaxation (T2) [16]. This experimental protocol was described and optimized by the Institute of Nuclear Sciences Applied to Health, ICNAS, University of Coimbra, Portugal [17]. Lipid mass fraction and fatty acid saturation were determined as previously described [18, 19]. Esterification percentage was calculated as the ratio between the number of glycerol carbons (5.19 ppm) and esterified fatty acids (2.24 ppm). The ratio between glycerol molecules and total number of fatty acids (1/3∗0.9 ppm) was also calculated, as a marker of increased probability of the existence of nonesterified fatty acids. For all measurements, a fit error less than 5% was used as a quality criterion.

### 2.7. Blood and Liver Collection

Animals were anesthetized, and serum and plasma were collected as described before [11, 12]. After sacrifice by cervical displacement, liver was photographed (Zeiss, Germany) and tissue samples were frozen (-80°C) or stored in 10% formalin.

### 2.8. Blood Analyses

Plasma levels of FFA and insulin were assessed using the FFA Assay Kit (ZenBio, NC, USA) and the Rat Insulin ELISA Kit (Mercodia, Sweden) (*n* = 8). Serum adiponectin levels were determined using the Rat Adiponectin Immunoassay Kit (Invitrogen, USA) (*n* = 8). Plasma levels of triglycerides, total cholesterol, HDL cholesterol, total protein, albumin, alanine aminotransferase (ALT), aspartate aminotransferase (AST), alkaline phosphatase, gamma-glutamyl transferase (GGT), and total bilirubin were measured in an automatic analyser at Clinical Pathology Service, Centro Hospitalar Universitário de Coimbra, Portugal (*n* = 8).

### 2.9. GLO-1 Activity

Liver samples (50 mg) were homogenized in 25 mM Tris-HCL, 10 mM NaCl buffer, pH = 7.4, and diluted 20x. GLO-1 activity was determined using the Glyoxalase 1 Activity Assay Kit (Sigma-Aldrich, MO, USA) (*n* = 6).

### 2.10. Western Blotting

Liver (100 mg) (*n* = 5 − 6) was homogenized and assayed as before [11, 12]. The secondary antibodies were anti-mouse (GE Healthcare, UK), anti-rabbit, and anti-goat (Bio-Rad, USA). Membranes were revealed using ECL substrate in a VersaDoc system (Bio-Rad, USA) and analysed with ImageQuant® (Molecular Dynamics, USA).

### 2.11. Histology

Tissue sections (4 *μ*m) from paraffin-embedded liver (*n* = 3/group) were stained with hematoxylin-eosin. Images were captured in a Zeiss microscope with an incorporated camera (Germany).

### 2.12. Dihydroethidium (DHE) Staining

For evaluation of superoxide anion staining through a DHE probe, tissue sections (8 *μ*m) from cryopreserved livers (*n* = 4/group) were fixed in chilled methanol, incubated during 30 minutes in 5 *μ*M dihydroethidium (Molecular Probes, USA), and mounted in an aqueous mounting medium. DAPI was used for the counter-staining of the nuclei. Images were captured in a fluorescence Zeiss microscope with the incorporated camera (Germany).

### 2.13. Gas Chromatography, TLC, and HPLC-MS Analysis of Lipid Extracts

Liver tissue (*n* = 6) was homogenized in phosphate buffer (PBS), pH 7.4, and lipid extraction was performed using the Folch method [20], with a chloroform : methanol (2 : 1 *v*/*v*) solution.

Total phospholipid (PL) quantification was measured by colorimetric phosphorous assay, as described before [21]. Phospholipid content per class was obtained by thin layer chromatography as previously described [22].

For phospholipid (PL) detection, samples (20 *μ*g of total phospholipid) were separated by HPLC as previously described [22] (HPLC; Waters Alliance 2690 with Ascentis Si column), which was coupled to a linear ion trap (LXQ; Thermo Finnigan, San Jose, CA, USA) mass spectrometer. The LXQ was operated in both positive (electrospray voltage +5 kV) and negative (electrospray voltage -4.7 kV) with 275°C capillary temperature and the sheath gas flow of 8 U. Normalized collision energy (CE) varied between 20 and 27 (arbitrary units) for MS/MS. Data acquisition was carried out on an Xcalibur data system (V2.0). Phospholipid classes were assessed in the negative ion mode, and data are presented by means of relative abundance per class: lysophosphatidylcholines (LPC), phosphatidylcholines (PC), sphingomyelins (SM), phosphatidylethanolamines (PE), cardiolipins (CL), phosphatidylserines (PS), phosphatidylinositols (PI), and phosphatidylglycerol (PG). MS/MS was performed for each ion to identify and confirm their structure, according to the typical fragmentation pathways [23], LIPID MAPS, and LIPID Mass Spec. Prediction program (v1.5, LIPID MAPS, 2009). Phospholipid internal standards were purchased from Avanti Polar Lipids Inc. (Alabaster, AL, USA).

Total esterified fatty acids were measured by gas chromatography–flame ionization detection (GC-FID) after transesterification of lipid liver extracts (approximately 90 *μ*g of total PL) using a gas chromatograph (Clarus 400, PerkinElmer Inc., USA) with a DB-1 column (J&W Scientific, Agilent Technologies, Folsom, CA, USA) as before [24]. Briefly, samples were prepared with a methanolic solution of KOH (2 M). The GC injection port was programmed at 523.15 K and the detector at 543.15 K. The oven temperature was programmed as follows: initially stayed for 3 minutes at 323.15 K, raised to 453.15 K (25 K min^−1^), held isothermal for 6 minutes, with a subsequent increase to 533.15 K (40 K min^−1^), and maintained there for 3 minutes. The carrier gas was hydrogen at 1.7 mL/min. C17 (7.5 *μ*g) fatty methyl ester was used as internal standard.

Esterified fatty acids from phospholipid fraction were determined after separation by thin layer chromatography for further analysis of the fatty acid profile. A volume of 30 *μ*L of each sample in chloroform (which contained an amount of 30 *μ*g of phospholipid) was applied on the TLC silica gel (Merck, Darmstadt, Germany) and eluted in a mixture of hexane/diethyl ether/acetic acid (80 : 20 : 1, by volume). The lipid spots were revealed under UV light (UV lamp 366 nm, CAMAG, Berlin, Germany) after spraying the air-dried plate with a solution of primuline (50 *μ*g mL^−1^) dissolved in water/acetone (20 : 80, by volume). The spots corresponding to phospholipids (PLs) and triacylglycerides (TGs), identified by comparisons with the migration of the spots with standards applied in the TLC plates, were scraped and extracted from silica with CHCl_3_ : MeOH : H_2_O (8 : 4 : 3). The solution was recovered after centrifugation at 1000 rpm for 5 min. The FA methyl esters (FAME) from the phospholipid pool were obtained and analysed by GC-FID as previously described.

### 2.14. Statistical Analysis

Results are presented as mean ± SEM (*n* = 6 − 8 per group). Given the small sample number, the nonparametric Kruskal-Wallis test (all pairwise multiple comparisons) was applied to determine statistical differences between the groups, using the SPSS software (IBM, NY, USA). *p* < 0.05 was considered significant.

## 3. Results

### 3.1. High-Fat Diet Leads to Body Weight Gain and Liver Steatosis while Glycation Triggers Portal Inflammation

HFD rats had increased body weight than controls (Ct group) and MG-supplemented rats (MG group). HFD rats with MG supplementation (HFDMG group) had a smaller increase in body weight (nonsignificant), despite the fact that they had eaten the same amount of food as HFD rats. No differences were observed for MG rats (Table 1). As well, no significant differences were observed for liver weight (Table 1) and plasma levels of the hepatic enzymes ASAT, ALAT, *γ*GT, and alkaline phosphatase (data not shown) in all groups. However, HFDMG rats had lower levels of plasma albumin levels, an unspecific marker of liver dysfunction, when compared to controls and MG rats (Table 1).

**Figure 1:**
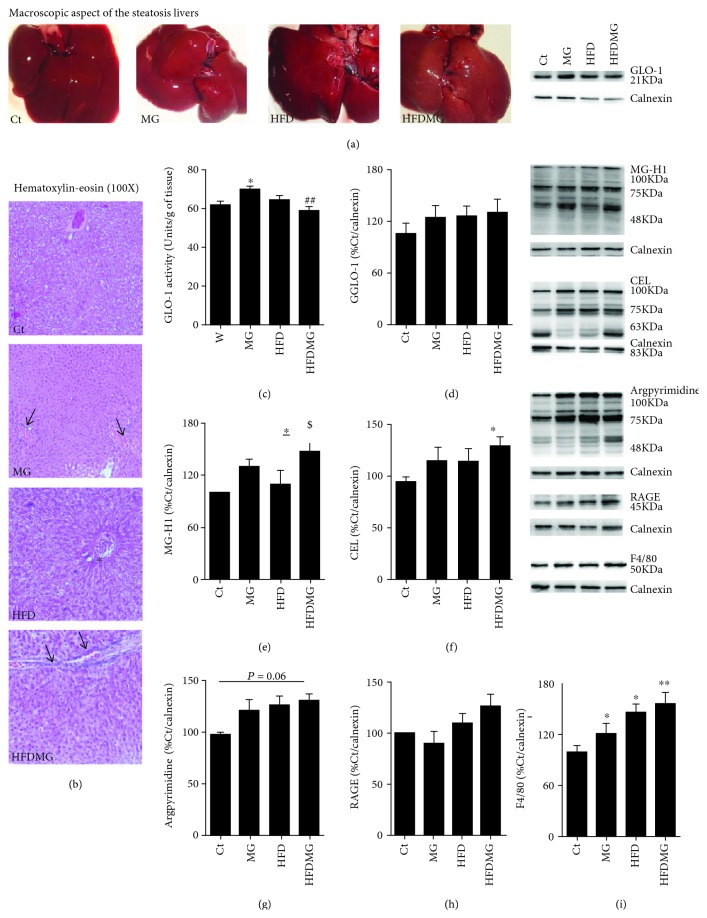
Macroscopic steatosis was observed in the livers from HFD and HFDMG groups (a), and the results were confirmed by histological analysis (b). However, portal inflammation (black arrows) was observed only in MG-treated rats (MG and HFDMG groups), especially in the combination group (100x). No portal inflammation was observed in the HFD group (black asterisk). GLO-1 activity was increased in MG-supplemented rats but not in HFDMG rats (c),while no changes were observed in the levels of the protein measured by Western blot (d). Increased liver levels of MG-H1 (e) and CEL (f) in HFDMG rats, calculated as percentage of Ct, while no significant alterations were observed for argpyrimidine (g) and RAGE (h). HFD rats show increased levels of the macrophage membrane marker F4/80, which is further increased by the enhanced glycation in obese rats (i). Representative WB are shown. Ct: Wistar 12 m; MG: Wistar + MG supplementation; HFD: HF diet-fed Wistar; HFDMG: HF diet-fed Wistar + MG supplementation. Bars represent means ± SEM. ∗ vs. Ct. 1 symbol *p* < 0.05; 2 symbols *p* < 0.01.

Macroscopic steatosis was confirmed by histological analysis, with microvesicular steatosis observed in both groups maintained with high-fat diet (HFD and HFDMG) (Figures 1(a) and 1(b)). However, only HFDMG rats had portal inflammatory infiltration, which was also partially observed in MG rats (Figure 1(b)). Such observations were consistent with F4/80 levels in the liver, a membrane marker of macrophages and Kupffer cells, which were higher in HFDMG rats (Figure 1(i)). In order to assess the accumulation of glycated products in the liver, the levels of AGE directly formed from MG MG-H1, CEL, and argpyrimidine were assessed by Western blot. Liver levels of MG-H1 and CEL were significantly higher in HFDMG rats than in controls, while argpyrimidine levels did not significantly differ between groups (*p* = 0.06) (Figures 1(e)–1(g)). No significant differences were observed in the membrane RAGE isoform (Figure 1(h)). Although no differences were also observed for GLO-1 expression, the key limiting enzyme involved in MG detoxification, its activity was significantly increased in the liver of MG rats but significantly decreased in HFDMG rats (Figures 1(c) and 1(d)).

### 3.2. Glycation Decreases Cytoplasmic Lipid Esterification and Unsaturation in Fatty Livers without Changing Total Lipids

**Figure 2:**
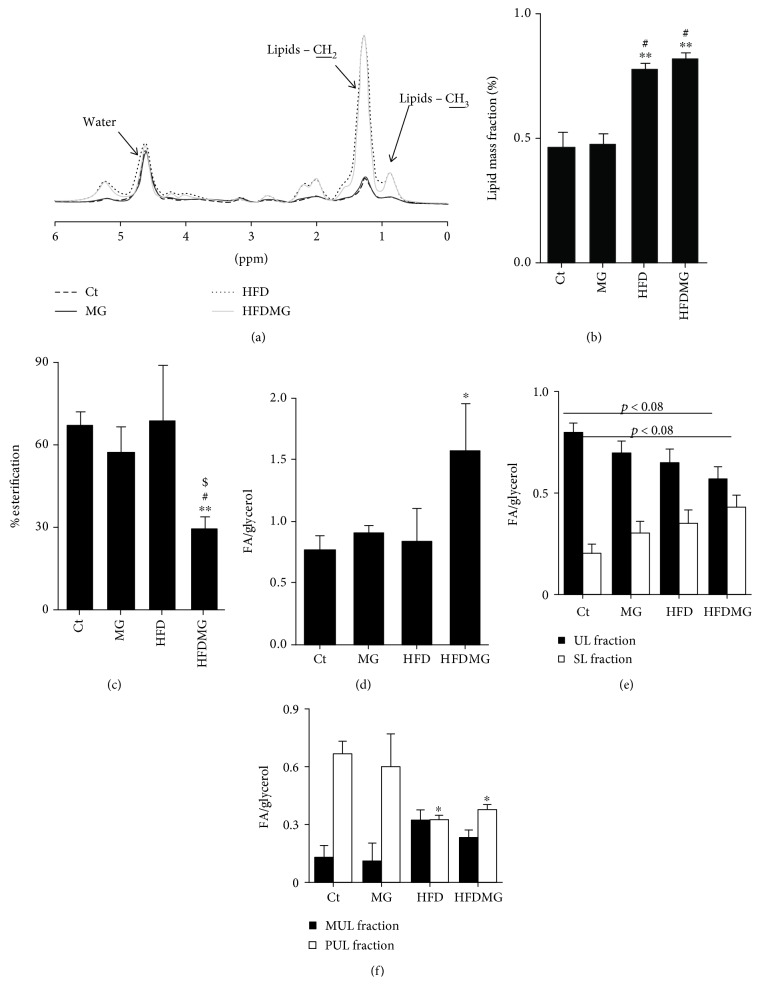
MRS shows increased the non-membrane lipid mass fraction after the consumption of a high-fat diet (HFD) and HFDMG groups (b). Complete spectra with average values/group show a similar increase in both groups of protons from the end of the chain (CH3) and in the middle of the chain (CH2), while there were no changes in water (a). MRS also showed a decreased percentage of esterification (c) and increased fatty acid/glycerol ratio (d) only in HFDMG rats. MRS analysis also revealed increased saturation in HFDMG rats (e), as well as decreased polyunsaturation in HFD and HFDMG (f). Ct: Wistar 12 m; MG: Wistar + MG supplementation; HFD: HF diet-fed Wistar; HFDMG: HF diet-fed Wistar + MG supplementation. Bars represent means ± SEM. ∗ vs. Ct; # vs. MG; $ vs. HFD. 1 symbol *p* < 0.05; 2 symbols *p* < 0.01.

MRS detects the number of cytoplasmic fatty acids, i.e., not included in membranes, including nonesterified fatty acids, triacylglycerols, and diacylglycerols. MRS data showed increased lipid fraction in HFD and HFDMG rats' livers compared to controls and MG rats (spectra of mean values per group are shown in Figure 2(a)), and similar between them (Figure 2(b)). The percentage of occupied glycerol carbons was determined by MRS using the ratio between the number of esterified fatty acids and the number of glycerol carbons. Moreover, the ratio between the number of total fatty acids and glycerol molecules was also determined. Decreased esterification of glycerol carbons and increased ratio between the total number of fatty acids and glycerol were found only in the HFDMG group, when comparing to all the other groups (Figures 2(c) and 2(d)).

**Figure 3:**
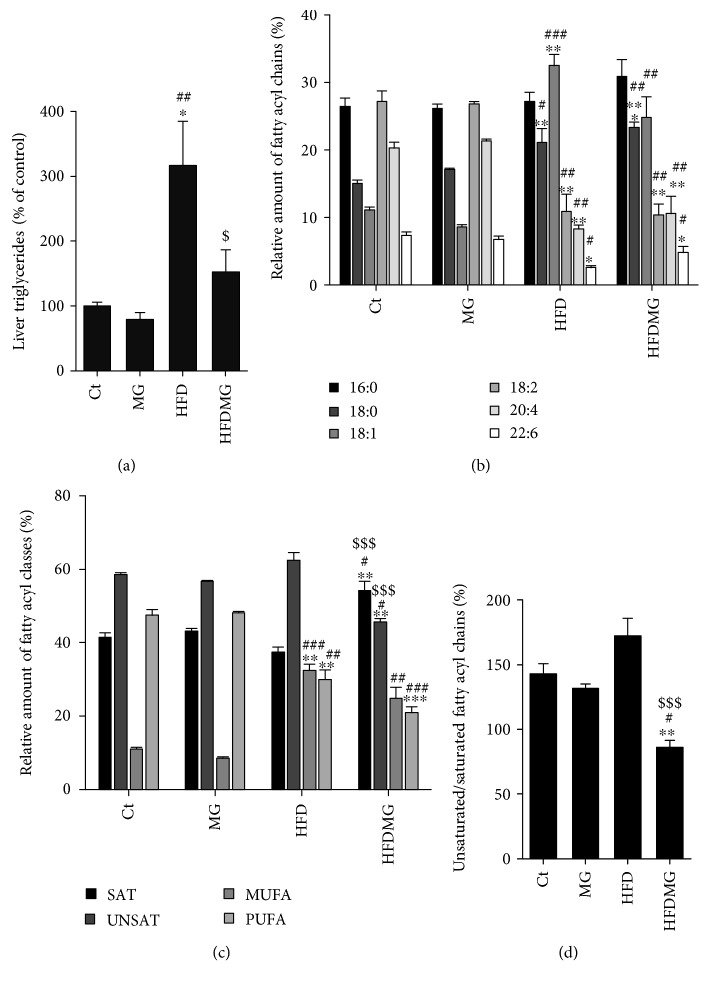
GC-FID analysis of FAME shows increased levels of esterified fatty acids in HFD, but not in HFDMG rats (a). Determination of fatty acyl methyl esters by GC-FID revealed increased 18 : 1 levels in HFD but not in HFDMG rats, as well as a further increase of 18 : 0 in HFDMG rats and decreased polyunsaturated fatty acids in both groups (b). Increased total saturated fatty acyl chains and decreased monounsaturated fatty acyl chains were observed in the HFDMG group (c), as well as decreased unsaturated/saturated ratio (d). Ct: Wistar 12 m; MG: Wistar + MG supplementation; HFD: HF diet-fed Wistar; HFDMG: HF diet-fed Wistar + MG supplementation. Bars represent means ± SEM. ∗ vs. Ct; # vs. MG; $ vs. HFD. 1 symbol *p* < 0.05; 2 symbols *p* < 0.01; 3 symbols *p* < 0.001.

MRS is able to quantify the number of double bonds in total non-membrane fatty acids. MRS demonstrated a decreased fraction of total non-membrane unsaturated fatty acids in HFDMG rats (*p* < 0.08) and lower levels of polyunsaturated fatty acids in HFD and HFDMG rats (Figures 3(e) and 3(f)). Such results demonstrate that dietary glycotoxins increase lipid saturation, particularly decreasing monounsaturated fatty acids.

### 3.3. Glycation Decreases Esterified and Unsaturated Fatty Acyl Chains in the Liver

GC-FID analysis of fatty acyl methyl esters (FAME) allows detecting total esterified fatty acids in the sample, including those incorporated into phospholipids. While the HFD group had significantly higher levels of esterified fatty acids compared with controls and the MG group, such increase was not observed in the HFDMG group (Figure 3(a)). Together with MRS analysis showing a similar increase in lipid fraction and lower percentage of esterification, such results suggest that glycation does not change total lipid levels in the liver but significantly decreases their esterification.

GC-FID detected increased levels of monounsaturated (18 : 1) and decreased saturated and polyunsaturated fatty acids in the pool of esterified fatty acyl chains in the HFD group, which is in accordance with the high levels of monounsaturated fatty acids in the diet (Figure 2(b)). Glycation caused a similar decrease in polyunsaturated fatty acids but not an increase in monounsaturated fatty acids, specifically 18 : 1 (Figures 2(b) and 2(c)), despite that both groups have eaten the same amount of food (Table 1). Moreover, glycation further increased the levels of 18 : 0 and total saturated fatty acyl chains in the HFDMG group, resulting in decreased unsaturated/saturated ratio and suggesting that glycation decreases fatty acid unsaturation specifically in the context of fatty liver (Figures 2(b)–2(d)).

### 3.4. Glycation Decreases Hepatic Polyunsaturated Cardiolipins and Increases Superoxide Anion Staining

**Figure 4:**
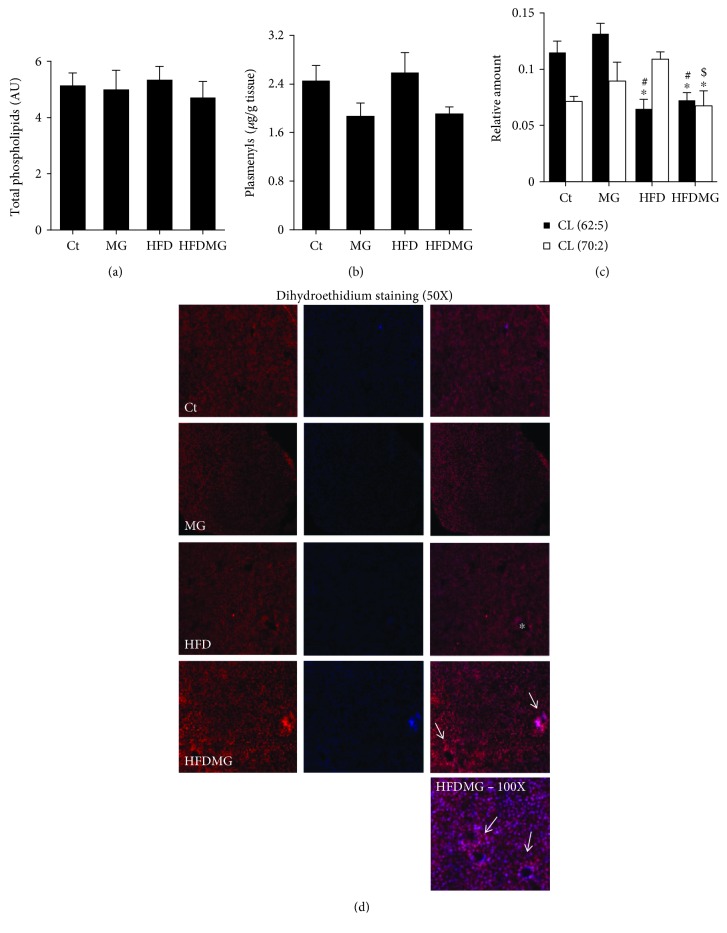
Total phospholipid content in the liver similar in all experimental groups (a). Rats supplemented with MG had a trend to reduced total plasmenyls levels (b), while its supplementation specifically to obese rats (HFDMG group) has led to a further decrease in polyunsaturated cardiolipins (d). Oxidative stress was confirmed through dihydroethidium (DHE) staining. (i) shows DHE staining (red), nuclei staining using DAPI (blue), and merged images. DHE was used to stain superoxide anion, and increased reactivity was observed in HFDMG liver sections (50x), especially in the portal spaces (white arrows). The bottom image shows a section of an HFDMG liver (100x) with increased reactivity around the portal spaces (white arrows). No reactivity was found in the same area of livers from obese rats without glycotoxin supplementation (white asterisk). Ct: Wistar 12 m; MG: Wistar + MG supplementation; HFD: HF diet-fed Wistar; HFDMG: HF diet-fed Wistar + MG supplementation. Bars represent means ± SEM. ∗ vs. Ct; # vs. MG; $ vs. HFD. 1 symbol *p* < 0.05.

Given the differences between MRS and GC-FID in the analysis of the different lipid pools in the cell, the levels and fatty acyl composition of phospholipids were assessed. The total levels of phospholipids in the liver, calculated after separation of PL classes by LC-MS, were similar in all groups (Figure 4(a)). As well, no differences were observed in the total levels of each phospholipid class (lysophosphatidylcholines (LPC), phosphatidylcholines (PC), sphingomyelins (SM), phosphatidylethanolamines (PE), cardiolipins (CL), phosphatidylserines (PS), phosphatidylinositols (PI), and phosphatidylglycerol (PG)), determined by TLC (data not shown). As well, after phospholipid separation by TLC and fatty acid analysis by GC-FID, no significant changes were observed in their composition in fatty acyl chains (data not shown), suggesting that changes occur at the cytoplasmic level, as shown in MRS analysis. Beyond the changes of total phospholipids, changes in specific phospholipid species may reveal alterations of cellular physiology and specifically increased oxidative damage. Although no changes were observed in the total amount of phospholipids and their classes, data from LC-MS analysis of the total lipid extract allowed pinpointing a trend for reduction of total plasmenyls (antioxidant phospholipids) in both groups supplemented with MG, although no significant differences were achieved (Figure 4(b)). Importantly, a significant reduction was observed in two specific polyunsaturated cardiolipins (mitochondrial phospholipids), most probably due to increased oxidative stress. While cardiolipin 62 : 5 was significantly decreased in both groups maintained with the high-fat diet, the levels of cardiolipin 70 : 2 were specifically decreased only in the HFDMG group, suggesting increased oxidative damage in the liver of obese rats supplemented with dietary glycotoxins (Figure 4(c)). In order to address this hypothesis, dihydroethidium (DHE) staining was used to evaluate the formation of superoxide anion. DHE has shown more reactivity in HFDMG rats, suggesting increased levels of oxidative stress in such rats. Importantly, increased DHE staining was particularly observed in portal regions, which is consistent with increased portal inflammation observed in the same experimental group (Figure 1(h)).

### 3.5. Glycation Increases Plasma Free Fatty Acids, Dysregulates Hepatic Pathways of Lipid Synthesis, and Causes Insulin Resistance

**Figure 5:**
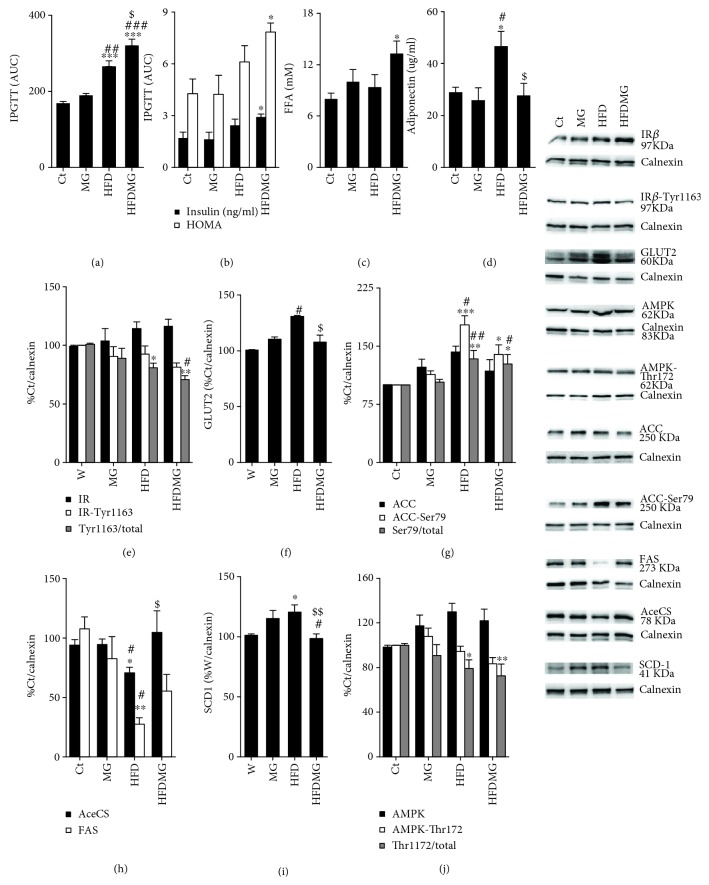
The consumption of a high-fat diet increased glucose intolerance (a), but MG supplementation further increased glucose intolerance and increased plasma insulin levels and HOMA (b). Plasma free fatty acid levels were significantly elevated in HFDMG rats (c). As well, glycation also prevented the hyperadiponectinemia observed in HFD rats (d). In the liver, the high-fat diet caused a decrease in insulin receptor activation (e), which was further increased by glycation, as well as a compensatory increase in GLUT2 (f), which was prevented by glycation. HFD rats had inhibition of key enzymes of *de novo* lipogenesis as ACC, AceCS, and FAS and upregulation of the desaturation enzyme SCD-1, which was not observed in HFDMG rats (g, h, i). Decreased AMPK activation was also observed in HFDMG rats (j). Intensity was calculated as percentage of Ct; representative WB are shown in the right panel. Ct: Wistar 12 m; MG: Wistar + MG supplementation; HFD: HF diet-fed Wistar; HFDMG: HF diet-fed Wistar + MG supplementation. Bars represent means ± SEM. ∗ vs. Ct; # vs. MG; $ vs. HFD. 1 symbol *p* < 0.05; 2 symbols *p* < 0.01; 3 symbols *p* < 0.001.

The consumption of a triglyceride-enriched diet resulted in increased glucose intolerance, i.e., area under the curve (AUC), during the intraperitoneal glucose tolerance test, but no significant alterations in insulinemia and HOMA (Figures 5(a) and 5(b)). Regarding MG supplementation to standard diet, no differences were observed. On the other hand, HFDMG rats had hyperinsulinemia, increased HOMA levels, and further increased AUC, being significantly increased when compared to the HFD group (Figures 5(a) and 5(b)). In the liver, no differences were observed in the MG group regarding insulin signaling, while the consumption of the triglyceride-enriched diet (HFD group) caused a decrease in phosphorylated/total insulin receptor ratio and a compensatory increase in GLUT2. The supplementation of MG to the HF diet caused a further decrease in phosphorylated/total insulin receptor ratio and inhibited GLUT2 increase, showing that glycation-driven changes in the liver lipidemic profile are associated with impaired insulin receptor signaling and contributes to insulin resistance in obesity (Figures 5(e) and 5(f)).

Given the changes in liver lipidemic profile in the liver of HFD-induced obese rats with MG supplementation, the levels of key enzymes in fatty acid oxidation, desaturation, and synthesis were determined. HFD rats had normal levels of triglycerides (Table 1) and free fatty acids, increased adiponectinemia, decreased AMPK activation, suppression of lipid synthesis pathways, and upregulation of the desaturation enzyme SCD-1 in the liver (Figures 5(c)–5(j)), while MG alone did not result in any alterations. On the other hand, MG supplementation to the high-fat diet caused increased levels of free fatty acids when compared to controls, lower levels of adiponectin levels than HFD rats (Figures 5(c) and 5(d)), and lower suppression of lipid synthesis pathways. In particular, when compared to the HFD group, HFDMG rats had lower phosphorylation (inactivation) of ACC and increased expression of FAS and AceCS, key enzymes in free fatty acid synthesis (Figures 5(g) and 5(h)). As well, phosphorylated (activated) AMPK levels were further lowered in HFDMG rats (Figure 5(j)). Regarding fatty acid saturation, levels of SCD-1, the main enzyme involved in fatty acid desaturation, were increased in the HFD group but not in the HFDMG group (Figure 5(i)). Such results suggest that glycotoxins impair the inhibition of lipid synthesis pathways and downregulate oxidation and desaturation pathways, possibly contributing to increased levels of nonesterified and saturated fatty acids in liver and plasma.

## 4. Discussion

The initial idea that NAFLD would be the liver manifestation of the metabolic syndrome is currently changed, and it is believed that it may in fact precede metabolic syndrome and type 2 diabetes [2, 25, 26]. Recent evidences suggest NAFLD as an independent risk factor for metabolic syndrome features and type 2 diabetes, with insulin resistance being the target player [2, 27–29]. In this study, we hypothesized that dietary glycotoxins may change the liver lipidemic profile and consequently insulin resistance in obesity. In order to assess that, we developed an animal model with diet-induced obesity and methylglyoxal-induced glycation. We used different and complementary techniques, magnetic resonance spectroscopy, GC-FID, and HPLC-MS in order to evaluate the lipidemic profile. MRS is one of the most accurate among the noninvasive diagnostic methods for NAFLD and is useful for noninvasive early diagnosis in human subjects [30]. Despite being less sensitive than biochemical analysis, it informs about all the lipid classes out of cellular and organelle membranes, including their saturation and esterification. On the other hand, GC-FID is more accurate in the evaluation of the lipidemic profile but requires invasive sample collection and, depending on the derivatization method used, it can only detect esterified lipids, missing the fatty acids of the NEFA pool [31]. In this study, we compare the results obtained with each of them and show their complementarity in the evaluation of the alterations of the liver lipidemic profile caused by dietary glycotoxins.

Hepatic lipids are mostly originated from the diet, *de novo* lipogenesis, and adipose tissue lipolysis [3]. Although adipose tissue is the physiological local to store fatty acids, avoiding their deposition in other tissues, insulin resistance is the main trigger to lipolysis and consequent elevation of plasma FFA, as well as their ectopic deposition in liver and muscle [32–35]. Interestingly, the same animal model described in this study also develops adipose tissue insulin resistance [15], which may contribute to the increased fatty acid flux to the liver. While accumulation of TG-enriched lipid droplets in the liver is considered harmless and suggested to protect from fatty acid-induced insulin resistance, ectopic nonesterified fatty acids are known to be a trigger to inflammation and insulin resistance [2, 36]. Moreover, Xia and colleagues recently demonstrated the involvement of ceramides in the development of hepatic insulin resistance and described that increased ceramide degradation reduced hepatic steatosis via inhibition of inflammation [37, 38]. Such types of lipids activate F4/80-expressing Kupffer cells, which create the inflammatory environment to NAFLD progression to NASH. Here, we show that accumulation of glycated products leads to the increase in total liver F4/80 levels and portal inflammatory infiltration.

The investigation about the involvement of dietary saturated and unsaturated fatty acids in liver fat accumulation has been extensive. MUFA and omega-6 PUFA were shown to decrease liver fat, while saturated fatty acids are associated with increased liver fat accumulation (reviewed by Marchesini et al. [3] and Hardy et al. [39]). Accordingly, dietary replacement of saturated fatty acids by omega-3 PUFA without changing the total calories was shown to improve NAFLD progression to NASH through decreasing lipid peroxidation and inflammation [40–43]. Such effects probably derive from different regulations of lipid oxidation machinery, as shown by Priore and colleagues, who demonstrated impaired lipid oxidation in rats fed a saturated fatty acid-enriched diet [44]. Here, we demonstrate by GC-FID that the consumption of a high-triglyceride diet leads to decreased relative levels of PUFA (20 : 4 and 22 : 6) in the total liver esterified lipids, due to increased MUFA levels, namely, 18 : 1, which is likely to be caused by the high levels of this MUFA in the diet. Glycation inhibited the increase in total 18 : 1 and MUFA levels in the liver, with a similar decrease in PUFA. Overall, total unsaturated fatty acyl chains were further decreased in the HFDMG group, showing that glycation increases lipid saturation, especially by decreasing MUFA. Importantly, a similar increase in saturated fatty acids was observed in total non-membrane fatty acids by MRS. Moreover, our results also demonstrate that such changes do not occur at the level of phospholipids, which is in accordance with MRS data, which do not detect membrane lipids.

Recently, Duarte and colleagues demonstrated suppression of *de novo* lipogenesis but not fatty acid esterification in mice fed a high-fat diet [37, 38]. Accordingly, our results also show that rats fed a high-triglyceride diet have inhibition of key enzymes of fatty acid synthesis (ACC, FAS, and AceCS) and increased levels of esterified lipids in the liver. Thus, in physiological conditions, the liver has the ability to inhibit fatty acid synthesis and store the ones coming from the diet or adipose tissue in nonharmful stores [37, 38, 45]. However, glycation blocked ACC, FAS, and AceCS suppression, leading to increased NEFA levels and thus to the activation of inflammatory pathways. Recent data from Gugliucci have shown increased hepatic lipogenesis after fructose feeding through direct inactivation of AMPK by fructose-derived MG [46]. Accordingly, in the study from Mastrocola and colleagues, an AGE-induced increase in lipogenesis via SREBP-1 activation was also observed, which is in accordance with our results [47]. Several studies have shown increased lipogenesis after exposure to AGE, which was not observed in our study in standard diet-fed rats. However, such studies were performed in cell cultures and thus it is not easy to compare to results obtained *in vivo*; besides, they have used distinct AGE dosages and methods of preparation [48–51].

The effects of glycation may result from increased oxidative stress generation and impaired mitochondrial function, as shown by decreased AMPK activation and polyunsaturated cardiolipin levels, a marker of mitochondrial oxidative stress and mitDNA damage [52, 53]. Our results demonstrate that even without major changes in total levels of the different phospholipid classes, a specific decrease in polyunsaturated cardiolipins is further observed in obese rats supplemented with MG. Despite that no significant differences were observed, the trend observed for decreased total antioxidant phospholipids is in accordance with previous data showing MG-induced impairment of antioxidant systems [54]. Since GLO-1 activity is dependent on antioxidant systems (GSH), the decrease in its activity and the increased DHE staining to superoxide anion observed in HFDMG rats further suggests the impairment of antioxidant systems (reviewed by [7]).

AGE have been implicated in the development and progression of diabetic complications, but given that their levels are increased in obesity and prediabetes and several sugar-enriched foods, they have also been progressively implicated in the onset of the metabolic syndrome and insulin resistance. Several studies have demonstrated that the consumption of an AGE-enriched diet results in increased liver oxidative stress, fibrosis, and inflammation [9, 55, 56]. Recently, Gaens and colleagues described increased secretion of inflammatory mediators in hepatocytes associated with increased CML formation. In obese patients, they have also described a correlation between CML levels and the grade of hepatic steatosis and inflammation [57]. Accordingly, serum AGE levels have been suggested to function as biomarkers of NAFLD [58, 59]. Besides being formed as a by-product of glucose and fructose metabolism, methylglyoxal is also often found in sugar-rich foods with a high glycemic index, as high-fructose corn syrup (HFCS), which is commonly used in soft drinks (reviewed by [7, 60–62]). The consumption of HFCS has been associated with metabolic syndrome components, including increased liver fat accumulation and insulin resistance, mainly through increased *de novo* lipogenesis [3, 61]. Thus, the existing studies suggest that increased AGE accumulation in the liver may in fact contribute to the onset of NAFLD. Nevertheless, such effects have been shown to be RAGE-mediated and the pharmacological use of a soluble RAGE isoform was shown to exert protective effects in patients with NAFLD [63–65]. Here, we demonstrate for the first time using MRS, GC-FID, and HPLC/MS that dietary glycotoxin supplementation and hepatic AGE accumulation cause glycoxidative stress and dramatically disrupt lipid metabolism in obesity, including impaired suppression of *de novo* lipogenesis and increased levels of saturated and nonesterified fatty acids. Future studies are necessary in order to fully understand glycation-induced alterations in fatty acid metabolism, namely, the role of endogenous sources of MG and the contribution of NEFA, triglycerides, diacylglycerols, or cholesterol ester pools, to the alterations observed in the levels of each fatty acid. Accordingly, the fatty acid content of plasma cholesterol esters was found to be significantly correlated with body fat depots and insulin resistance [66, 67]. Nevertheless, the mechanisms here described may be involved in lipotoxicity-driven hepatic inflammation and impairment of glucose metabolism, contributing to NAFLD pathogenesis and progression to NASH.

This study demonstrates for the first time the changes in lipid metabolism in obesity occurring by diet-induced liver AGE accumulation. In accordance with previous studies suggesting the positive impact of anti-AGE therapies in NAFLD [65], our results suggest that decreasing dietary AGE may be a promising strategy in the prevention of NAFLD and its progression to NASH and open new possibilities to the development of new therapeutic strategies based on reduction of dietary AGE or modulation of detoxification systems. Importantly, the methods here used can be directly translated to human studies and thus the results here presented open new opportunities in finding new risk markers and therapeutic strategies in order to prevent NAFLD development and progression to NASH.

## Figures and Tables

**Table 1:** Food intake, body and liver weight, plasma total proteins, and albumin, serum lipids, and fasting glycemia.

Group	Ct	MG	HFD	HFDMG
Food (g/rat/day)	22.9 ± 0.7	24.3 ± 1.6	15.1 ± 1.1∗∗^##^	14.4 ± 0.7∗∗^##^
Weight (g)	508.8 ± 11.4	508.9 ± 18.4	652.7 ± 35.8∗∗^##^	571.6 ± 27.3
Liver weight (g)	13.9 ± 0.6	12.4 ± 0.6	13.4 ± 0.7	13.9 ± 0.7
Total plasma proteins (mg/dL)	6.4 ± 0.1	6.2 ± 0.1	6.0 ± 0.2	6.1 ± 0.1
Plasma albumin (mg/dL)	2.81 ± 0.1	2.85 ± 0.1	2.62 ± 0.1	2.47 ± 0.1∗∗^#^
Triglycerides (mg/dL)	75.4 ± 5.5	69.3 ± 10.7	77.8 ± 5.4	62.3 ± 3.2
Total cholesterol (mg/dL)	75.1 ± 4.6	74.3 ± 2.7	99.2 ± 9.4∗	91.7 ± 8.8
HDL cholesterol (mg/dL)	44.5 ± 2.7	45.8 ± 1.4	58.2 ± 4.7∗	55.7 ± 5.1
Total/HDL cholesterol	1.69 ± 0.1	1.62 ± 0.1	1.70 ± 0.1	1.66 ± 0.1
Fasting glycemia (mg/dL)	68.5 ± 2.0	70.6 ± 1.4	70.9 ± 2.0	71.1 ± 1.6

Ct: Wistar 12 m; MG: Wistar + MG supplementation; HFD: HF diet-fed Wistar; HFDMG: HF diet-fed Wistar + MG supplementation. Average ± SEM. ∗ vs. Ct; # vs. MG; $ vs. HFD; & vs. HFDMG. 1 symbol *p* < 0.05; 2 symbols *p* < 0.01; 3 symbols *p* < 0.001.

## Data Availability

All the data used to support the findings of this study are available from the corresponding author upon request.

## Additional Points

*Highlights*. High-triglyceride diet increases lipid content in liver and leads to suppression of *de novo* lipogenesis but not insulin resistance. Dietary glycotoxins do not change the liver lipid fraction of diet-induced obese rats but lead to higher fatty acid saturation and less esterification. Impairment of the lipidemic profile by glycotoxins is associated with the development of oxidative stress and insulin resistance in obese rats.
